# An Autonomous SAR Image Interpretation Algorithm Based on Multi-Agent Collaborative Scheduling

**DOI:** 10.3390/s26113311

**Published:** 2026-05-23

**Authors:** Dongdong Lu, Mingjie Zhang, Yibo Guo, Hang Li, Na Liu

**Affiliations:** 1 Suzhou Aerospace Information Research Institute, No. 158, Dushu Lake Avenue Suzhou Industrial Park, Suzhou 215000, China; yibo.guo@nuaa.edu.cn (Y.G.); lihang01@aircas.ac.cn (H.L.); 15062197213@163.com (N.L.); 2 Xi’an Aerospace Remote Sensing Data Technology Co., Ltd., National Civil Aerospace Industrial Base, Xi’an 710100, China; 18234097995@163.com; 3 Aerospace Information Research Institute, Chinese Academy of Sciences, Beijing 100094, China

**Keywords:** SAR image interpretation, MCP-based centralized collaborative scheduling, multi-agent systems, resource allocation, reinforcement learning

## Abstract

Synthetic Aperture Radar (SAR) image interpretation in dynamic scenarios faces critical challenges, including sluggish multi-agent scheduling responses, sub-optimal task-resource matching, and low full-pipeline collaborative efficiency. To address these issues, this paper proposes an autonomous SAR image interpretation algorithm based on a Mission Control Point (MCP)-driven centralized multi-agent collaborative scheduling framework. To address inefficient task–resource matching, a multi-source orchestration model integrating agent states, task characteristics, and environmental dynamics is developed for optimized initial allocation. To mitigate information fragmentation and improve collaboration efficiency across the pipeline, an MCP-based centralized architecture is proposed to achieve unified scheduling and global optimization of multi-stage agents. Furthermore, to enhance adaptability in dynamic environments, a verification-driven adaptive policy continuous optimization mechanism is introduced, allowing the scheduling policy to continuously adapt. Experiments have been conducted on the SARCAP public dataset, and the proposed method achieved a task–agent matching accuracy of 97.98%, an average scheduling latency of 66.1 ms, and a collaborative interpretation speed of 17.9 fps. Compared with MAPPO and conventional centralized scheduling, scheduling efficiency was improved by 12.3% and 18.7%, respectively. Ablation studies further indicate that both the MCP centralized scheduling mechanism and the multi-source information orchestration module significantly contributed to performance, ensuring high accuracy and robustness.

## 1. Introduction

Synthetic aperture radar (SAR) provides all-weather, all-time imaging capability with strong penetration and has become a core technology in airborne remote sensing for applications such as mapping, disaster response, military reconnaissance, and maritime monitoring [1,2]. As a mobile sensing platform, SAR can acquire high-resolution imagery under complex environmental conditions to support target detection, change detection, and object recognition tasks [3]. Particularly, advanced characterization of target scattering mechanisms [4] and robust maritime target detection under varying imaging geometries [5] have significantly pushed the boundaries of SAR interpretation accuracy. However, the dynamic nature of airborne platforms, together with constrained computational resources and environmental uncertainty, poses significant challenges to achieving autonomous, real-time, and high-precision SAR image interpretation. Multi-agent collaborative scheduling is a key component of SAR interpretation systems, where scheduling efficiency and task–resource matching accuracy directly affect system performance [6,7].

In remote sensing task scheduling, multi-agent approaches have attracted significant attention due to their capability for coordinated decision-making in dynamic environments. Recently, reinforcement learning (RL) and multi-agent reinforcement learning have been widely applied to complex scheduling problems, such as UAV task allocation [8], satellite constellation scheduling [9,10], and heterogeneous resource optimization [11]. Centralized strategies that integrate global information can effectively improve decision consistency and scheduling efficiency [12]. However, existing approaches often neglect computational and energy constraints, limiting their applicability in resource-constrained airborne systems [13]. In addition, traditional rule-based scheduling methods, such as first-come-first-served and round-robin, exhibit limited performance under highly dynamic and resource-competitive scenarios [14].

In the field of remote sensing image interpretation, multi-source information fusion has been explored for task scheduling and resource allocation. Existing studies integrate task priority, target importance, and resource availability to improve adaptive task assignment [15]. RL-based multi-source fusion strategies have demonstrated promising performance in satellite mission planning [16,17]. However, these methods mainly focus on single-stage planning or static optimization, which cannot meet the requirements of multi-stage collaborative scheduling in SAR interpretation pipelines.

Despite these advances, SAR interpretation systems still face significant challenges in multi-agent scheduling. Conventional approaches fail to jointly model device states, task requirements, and environmental dynamics, resulting in slow response and low matching accuracy [8,9]. Moreover, insufficient information exchange among different stages, including detection, preprocessing, interpretation, and intelligence generation, leads to low end-to-end coordination efficiency [10]. Existing algorithms also ignore computational and energy constraints and lack continuous optimization mechanisms to adapt to dynamic environments [13,14].

Multi-agent collaboration has achieved significant progress in artificial intelligence and has been widely applied in remote sensing, robotic coordination, and aerospace systems [16,18]. The centralized MCP (Mission Control Point) architecture provides a unified control paradigm that effectively eliminates information silos and supports full-chain scheduling [19]. Compared with distributed approaches, MCP offers advantages in global optimization and resource coordination. When combined with multi-source information fusion and reinforcement learning, it can further enhance adaptive scheduling capability [20]. However, under strict hardware constraints, existing approaches still exhibit limitations in adaptability and validation completeness.

To address these challenges, this paper proposes an MCP-based centralized multi-agent collaborative scheduling method for autonomous SAR image interpretation, which is validated on the SARCAP dataset. By integrating multi-source information orchestration and a verification-driven adaptive policy continuous optimization mechanism, the proposed method achieves efficient full-chain scheduling and continuous adaptation to dynamic airborne environments.

The main contributions of this work are summarized as follows:

- A multi-source information fused MCP centralized scheduling architecture that intrinsically circumvents the parameter redundancy and convergence bottlenecks typical of conventional centralized MARL frameworks (e.g., MAPPO). By leveraging a singular policy network alongside deterministic conflict resolution, this architecture enables highly efficient parameter sharing and coordinated decision-making across the full interpretation pipeline.
- A lightweight reinforcement learning-based task–resource orchestration algorithm tailored for edge computing constraints. It models device status, task characteristics, and environmental parameters into a unified state representation, achieving dynamic optimal matching through weighted fusion and a highly streamlined Proximal Policy Optimization (PPO) network.
- A verification-driven adaptive policy continuous optimization mechanism is constructed to establish a closed-loop optimization workflow consisting of performance monitoring, trigger evaluation, incremental fine-tuning, and rollback protection. The proposed mechanism employs dual-track performance monitoring to detect policy degradation and distribution drift in real time, and activates a hierarchical freezing-based fine-tuning strategy for rapid environmental adaptation. In addition, performance-driven model acceptance criteria together with historical best-policy rollback protection are adopted to ensure that system performance never degrades during online adaptation, thereby significantly enhancing the long-term robustness of the proposed algorithm in dynamic non-stationary environments.

The remainder of this paper is organized as follows. Section 2 introduces the system model and outlines the overall framework structure. Section 3 details the proposed scheme, including the multi-source data preprocessing pipeline, the task–resource orchestration algorithm, the MCP-based centralized collaborative scheduling architecture, and the adaptive policy continuous optimization mechanism. In Section 4, we provide a comprehensive discussion based on ablation studies. Finally, in Section 5, we outline the conclusions and future work.

## 2. System Model

In practical airborne SAR operations, onboard edge computing resources are intrinsically bottlenecked by stringent power and payload limitations. Consequently, executing real-time data processing necessitates algorithmic paradigms characterized by minimal memory footprints and attenuated computational overhead. The proposed framework systematically mitigates these physical restrictions through a synergistic structural design. Specifically, the multi-source data fusion module significantly compresses raw data dimensionality, while the integrated lightweight PPO policy network guarantees millisecond-level scheduling responsiveness. This architectural synthesis ensures that the overall system sustains optimal execution efficiency within the rigorous energy and processing envelopes inherent to edge-deployed airborne platforms.

**Figure 1:**
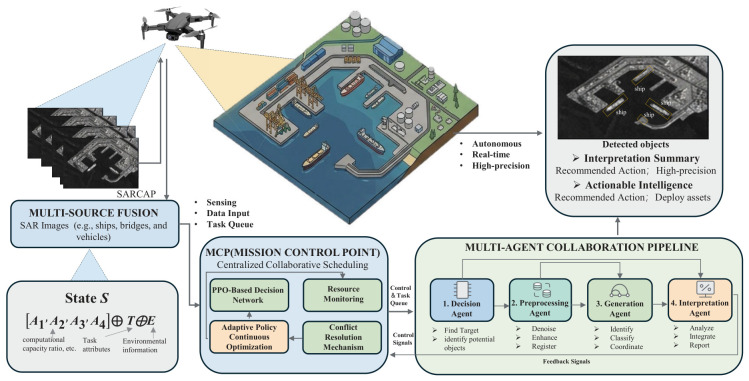
Illustration of the MCP-based multi-agent collaborative scheduling system for SAR image interpretation. Different colors in the figure are used to distinguish different functional modules.

To address the challenges of dynamic environments, limited onboard computational resources, and the low accuracy of task–resource matching, delayed scheduling response, and insufficient collaboration efficiency in SAR image interpretation, this paper develops a full-chain multi-agent collaborative scheduling framework based on PPO. The framework comprises four key modules, namely the overall architecture design, task–resource orchestration, MCP-based centralized scheduling, and adaptive policy optimization. This integration enables efficient collaborative scheduling among the four functional agents of detection, preprocessing, interpretation, and intelligence generation, as illustrated in Figure 1.

The framework takes multi-source heterogeneous data as input and constructs a globally observable state through a standardized preprocessing pipeline. A lightweight PPO-based policy network performs initial task–resource matching, generating a preliminary task–resource orchestration scheme.The MCP-based centralized scheduler executes global optimization, coordinating task allocation and execution pacing across the four functional agent types. An adaptive policy continuous optimization module establishes a closed-loop feedback, enabling real-time adaptation of model parameters to dynamic scenarios.

1. Multi-Source Data Fusion and State Construction: The framework utilizes multi-source heterogeneous data as input and constructs a globally observable state through a standardized preprocessing procedure. Environmental disturbance factors such as noise level, scene complexity, and motion stability are incorporated to simulate non-stationary conditions. The preprocessing produces a standardized ten-dimensional state vector that integrates real-time agent resources, task requirements, and environmental conditions and provides input for downstream task–resource orchestration.
2. Task–Resource Orchestration: The task-resource orchestration module implements a lightweight PPO policy network to perform preliminary task-resource matching. This provides an end-to-end mapping from state perception to resource allocation. A single centralized policy guarantees dynamic optimal matching between tasks and resources and forwards the allocation plan to the MCP centralized scheduler for global optimization.
3. MCP-Based Centralized Scheduling: The MCP scheduler forms the core of the framework and manages task queues and pipeline dependencies while coordinating the four agent types responsible for detection, preprocessing, interpretation, and intelligence generation. Resource monitoring continuously tracks agent load and a shared PPO network generates deterministic allocation decisions. Resource conflicts are resolved by a conflict resolution mechanism based on global priority scores. A feedback loop enables dynamic rescheduling at millisecond intervals and ensures efficient and stable multi-agent collaboration while overcoming limitations of distributed architectures such as isolated policies and lack of global observability.
4. Adaptive Policy Continuous Optimization: The adaptive policy continuous optimization module continuously monitors task matching accuracy and scheduling latency. Upon detecting performance degradation, an incremental learning mechanism is triggered. Base layer parameters are frozen while top layer parameters are fine-tuned to adapt to environmental changes. The optimized parameters are fed back to the task-resource orchestration module, establishing a closed-loop system encompassing orchestration, scheduling, execution, and optimization.

## 3. The Proposed Scheme

**Figure 2:**
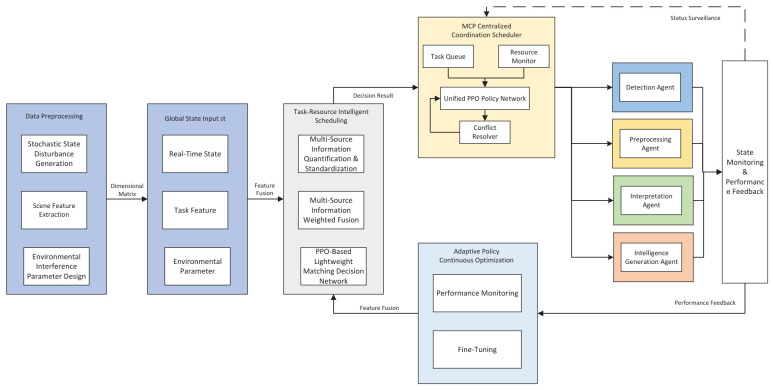
Overall algorithmic framework of the proposed MCP-based centralized multi-agent collaborative scheduling system. Different background colors are used to visually distinguish distinct functional modules, pipelines, and agent types.

In this section, we first describe the experimental dataset alongside the multi-source information preprocessing and quantification pipeline. Then, we provide the designs for the task–resource orchestration algorithm and the MCP-based centralized collaborative scheduling architecture. Finally, we present the adaptive policy continuous optimization mechanism designed to maintain robust performance in dynamic environments. The overall algorithmic framework of the proposed scheme is illustrated in Figure 2.

### 3.1. Experimental Dataset and Preprocessing

Experiments utilize the SARCAP dataset, which contains over 250 thousand image patches and 400 thousand image-text pairs. For target detection, the study focuses on the detection subset namely De-SAR, which includes targets such as ships, bridges, and vehicles. The dataset can be constructed for simulation and evaluation with reference to the De-SAR detection subset of the SARCAP dataset. Although the SARCAP dataset contains a large number of samples, many instances do not provide precise location annotations, while some images are associated with multiple textual descriptions, which makes them unsuitable for constructing standardized scheduling tasks in our framework. Accordingly, we generated 8000 training samples and 2000 testing samples for simulation and experimental evaluation. The constructed dataset comprehensively covers four representative SAR scenario categories while maintaining a balanced scenario distribution. Moreover, the distribution is highly consistent with the practical task proportions in real airborne applications. This design effectively alleviates scenario bias and further enhances the engineering relevance and practical reference value of the experimental results.

#### 3.1.1. Scene Feature Extraction and Quantification

Scene features are extracted from the textual descriptions of the SAR images using keyword matching algorithms to automatically identify the scene type. Scene complexity and computational resource requirements are quantified according to the expected processing load. Ports and wharfs require higher computational resources due to the presence of man-made structures, while open ocean scenes are simpler to process. The classification is summarized in Table 1.

#### 3.1.2. Task Attribute Representation

Task attributes are derived from semantic analysis of image descriptions and mapped to four agent categories. A priority-based assignment strategy is adopted to ensure that higher-value tasks are processed preferentially. In addition, task-specific accuracy requirements and latency constraints are defined according to functional characteristics and onboard computational limitations.

Detection tasks emphasize target presence recognition with relaxed accuracy requirements but high recall expectations. Preprocessing tasks require moderate accuracy to preserve image quality during enhancement operations. Interpretation tasks demand high precision for reliable target classification. Intelligence generation tasks operate at the decision-making level and require extremely high accuracy with strict latency constraints. The unified task attribute configuration is summarized in Table 2.

#### 3.1.3. Environmental Parameter Design

Environmental parameters are introduced to simulate non-stationary SAR operating conditions and evaluate the robustness of the scheduling strategy. These parameters are independently generated and decoupled from task semantics to avoid implicit label leakage.

Noise level represents the intensity of speckle noise and electromagnetic interference. Scene complexity reflects the statistical distribution of background clutter, ranging from homogeneous ocean surfaces to highly structured urban environments. Motion stability characterizes the stability of the airborne platform or target motion. The parameter ranges are summarized in Table 3.

#### 3.1.4. Standardized Data Structure

After preprocessing, the SARCAP detection subset is transformed into a standardized dataset suitable for multi-agent collaborative scheduling. Each sample contains image information, task attributes, and environmental parameters. The dataset schema is presented in Table 4.

### 3.2. Task–Resource Orchestration Algorithm

**Figure 3:**
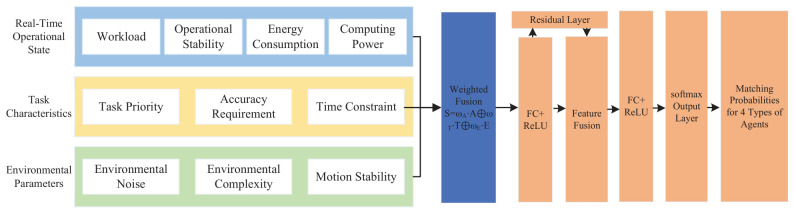
Architecture of the PPO-Based Lightweight Task-Resource Matching Decision Network. Different colors denote distinct types of input features and neural network processing layers.

The task–resource orchestration algorithm serves as the core component for achieving accurate matching between task demands and agent resources. Its objective is to construct an end-to-end decision mechanism from state representation to resource allocation by integrating multi-source heterogeneous information through unified quantification, weighted fusion, and lightweight policy learning. In this way, the proposed module improves task–resource matching accuracy, enhances adaptability to dynamic environments, and provides reliable initial decisions for the subsequent MCP-based centralized scheduler. All parameter settings and computational logic are determined according to engineering-oriented experimental optimization. The overall structure is illustrated in Figure 3.

Specifically, the input composition systematically integrates three distinct data streams encompassing the real-time operational state of the agents, prioritized task characteristics, and fluctuating environmental parameters. These heterogeneous inputs undergo a mathematically rigorous weighted fusion process to construct a comprehensively synchronized global state vector. This unified representation is subsequently ingested by the lightweight Proximal Policy Optimization decision network. To maintain low computational overhead while preserving feature depth, the network architecture sequentially processes the vector through fully connected layers equipped with ReLU activation functions, bolstered by a dedicated residual layer to effectively mitigate gradient degradation. Following a secondary internal feature fusion, the latent representations are fed into a softmax output layer. This final structural component efficiently maps the high-dimensional space into a normalized probability distribution, explicitly governing the matching suitability for the four distinct types of processing agents.

#### 3.2.1. Multi-Source Quantification and Normalization

To ensure effective fusion of heterogeneous information, the three categories of core inputs collected by the input module are first uniformly quantified and normalized. This process eliminates dimensional inconsistency and scale bias, thereby improving the stability and generalization capability of subsequent policy learning.

The agent resource state is represented by a four-dimensional normalized vector, which is defined as follows:

(1) $$A=[A_{1},A_{2},A_{3},A_{4}]$$

where $A_{1}$ denotes the remaining computational capacity ratio, $A_{2}$ denotes normalized energy consumption, $A_{3}$ denotes the task load ratio, and $A_{4}$ denotes operational stability. The initial state is defined as A=[0.5,0.5,0.5,0.5], ensuring consistent initial conditions across agents. The state can then be dynamically updated using sensor measurements to maintain real-time and reliable resource representation.

Task attributes are represented by a three-dimensional vector, which is defined as follows:

(2) T=[priority,accuracy_req,time_limit_ms]

where task priority can be discretized into low, medium, and high levels and mapped to numerical values with priority∈{1,3,5}, followed by normalization to [0,1]. The accuracy requirement and latency constraint are defined as accuracy_req∈[60,100] and time_limit∈[30,120] ms, and further normalized to [0,1]. To improve model generalization, explicit task category encoding is not introduced. Instead, the model learns the task–resource relationship from latent task attributes, including priority, accuracy, and latency, thereby avoiding direct overfitting to explicit labels.

Environmental information is represented by a three-dimensional vector, which is defined as follows:

(3) E=[env_noise,scene_complexity,motion_stable]

where env_noise∈[10,90], scene_complexity∈[20,95], and motion_stable∈[60,99]. All variables are further normalized to [0,1] to ensure a unified representation scale. This representation can comprehensively capture the external factors that affect SAR task execution and resource scheduling, thereby improving adaptability to complex airborne environments.

#### 3.2.2. Weighted Fusion of Multi-Source Information

After multi-source quantification, a fixed-weight fusion strategy is employed to construct a unified state representation. This strategy follows the principle that task demand dominates the scheduling decision while resource status and environmental effects can be considered. The optimal weight allocation can be determined through multiple rounds of engineering experiments to balance fusion effectiveness and computational efficiency. The fused state vector is defined as:

(4) $$S=\omega_{A}A⊕\omega_{T}T⊕\omega_{E}E$$

where ⊕ denotes vector concatenation, and $\omega_{A}$, $\omega_{T}$, and $\omega_{E}$ denote the fusion weights for resource state, task attributes, and environmental parameters, respectively. The weights satisfy $\omega_{A}=0.3$, $\omega_{T}=0.4$, and $\omega_{E}=0.3$. The fused state vector is given by $S\in R^{10}$. By integrating resource state, task attributes, and environmental parameters into a compact state space, this formulation retains essential heterogeneous information while reducing computational complexity for subsequent policy learning.

#### 3.2.3. Lightweight PPO-Based Matching Network

To achieve dynamic optimal matching between tasks and agent resources, a lightweight PPO-based policy network is constructed as the core decision model. The network is designed to balance matching accuracy with computational efficiency and training stability under onboard resource constraints. The policy is defined as a mapping function:

(5) $$\pi_{\theta }\left(S\right)=P$$

where $S\in R^{10}$ denotes the fused state vector and $P\in R^{4}$ denotes the assignment probability vector over the four agent types. The policy network is implemented as a lightweight feedforward architecture consisting of an input layer, a hidden layer with 64 neurons, and an output layer. Residual connections are introduced to strengthen feature propagation while maintaining low computational complexity. The output layer applies the Softmax function to produce the assignment probability distribution satisfying:

(6) $$\sum_{i=1}^{4}P_{i}=1$$

During forward propagation, the input state vector *S* is processed through both the main pathway and the residual pathway to obtain the action probability vector *P*. To transition from probabilistic network outputs to deterministic scheduling assignments, the system employs a rigid decision rule by extracting the dimension associated with the highest probability, mathematically formulated as:

(7) $$a^{*}=\arg\max_{i}P_{i}$$

where $a^{*}$ denotes the optimal scheduling assignment, and the argmax operation structurally guarantees that, within the physical context of the airborne interpretation pipeline, a specific interpretation task is strictly allocated to the agent exhibiting the highest instantaneous matching suitability, thereby effectively preventing execution ambiguity.

Policy optimization is performed using the PPO framework. The probability ratio is defined as $r_{t}=\frac{\pi_{\theta }\left(a_{t}|s_{t}\right)}{\pi_{\theta_{\mathrm{old}}}\left(a_{t}|s_{t}\right)}$. To ensure stable updates, a clipping mechanism is used to constrain the ratio within a bounded interval $r_{t}\in \left[1-\epsilon ,1+\epsilon \right]$ with ϵ=0.2, which effectively stabilizes training and prevents excessive policy deviation. In addition, LayerNorm is employed to improve convergence behavior and generalization performance under non-stationary environments.

The proposed network establishes an end-to-end mapping from multi-source fused states to task–resource allocation decisions. It can provide accurate and efficient matching results while maintaining low computational overhead, thereby serving as a critical bridge between multi-source information fusion and MCP-based centralized scheduling.

### 3.3. MCP-Based Centralized Collaborative Scheduling

**Figure 4:**
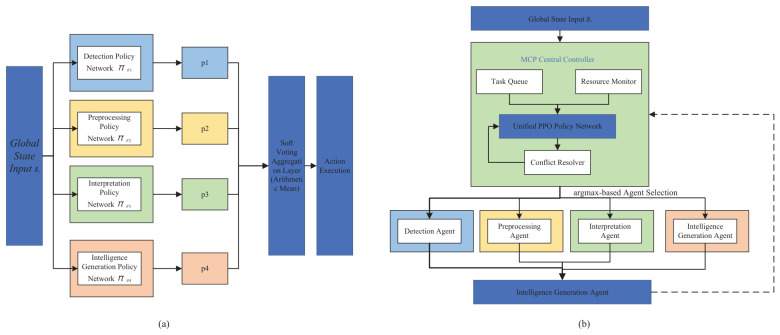
Comparison between Traditional Distributed Multi-Agent Architecture and MCP Centralized Collaborative Scheduling Architecture: (**a**) Traditional Distributed Multi-Agent Architecture. (**b**) MCP Centralized Collaborative Scheduling Architecture. Different background colors are used to visually distinguish distinct functional modules, policy networks, and agent types.

The multi-agent scheduling problem in SAR image interpretation can be formulated as a centralized partially observable Markov decision process. Under this formulation, the system performs unified decision making based on a globally observable state and updates the scheduling policy according to execution feedback. To explicitly elucidate the structural superiority of this approach, Figure 4 delineates a comprehensive comparison between the conventional distributed paradigm and the proposed Mission Control Point (MCP) framework. In the traditional distributed architecture, the global state input is divergently processed by isolated, task-specific policy networks. This configuration necessitates a heuristic soft-voting aggregation layer based on arithmetic means, which intrinsically restricts global optimality and exacerbates parameter redundancy. Conversely, the MCP framework consolidates decision-making authority within a singular central controller. This advanced orchestrator natively integrates real-time task queues and resource monitors to continuously inform a unified Proximal Policy Optimization network, thereby ensuring comprehensive global state sharing. Augmented by a dedicated conflict resolver, the centralized architecture executes deterministic, argmax-based agent selection while sustaining a robust closed-loop feedback trajectory from the execution layer to iteratively refine the shared policy.

To mathematically formalize the decision-making process executed by the aforementioned MCP orchestrator, the global state at time step *t* is denoted by $s_{t}\in S$. Within this formulation, $s_{t}$ comprehensively integrates the real-time task queue status, agent resource conditions, and environmental constraints monitored by the central controller. The corresponding deterministic scheduling action is defined as $a_{t}\in A$, representing the explicit assignment of interpretation tasks to the designated agents. Consequently, the unified scheduling policy is governed by $\pi_{\theta }\left(a_{t}|s_{t}\right)$, where θ precisely signifies the shared parameters of the central Proximal Policy Optimization network.

#### 3.3.1. Limitations of Distributed Scheduling

In conventional distributed architectures, multiple independent policy networks generate action probabilities $p_{i}=\pi_{\theta_{i}}\left(a_{t}|s_{t}\right)$. The final decision is obtained via aggregation $p=\frac{1}{N}\sum_{i=1}^{N}p_{i}$ with N=4. The formulation can suffer from policy homogenization, lack of global resource awareness, and inability to maintain pipeline dependencies, resulting in suboptimal scheduling performance.

#### 3.3.2. MCP-Based Centralized Scheduling

The Mission Control Point (MCP) functions as the overarching centralized orchestrator by executing continuous telemetry polling across the heterogeneous pool of detection, preprocessing, interpretation, and intelligence generation agents to sustain a globally synchronized resource state. This architecture is specifically designed to manage task queue maintenance, real-time resource monitoring, and seamless cross-agent collaboration, while establishing a robust closed-loop feedback mechanism with the strategy optimization module to sustain operational consistency. Furthermore, during instances of resource contention involving simultaneous task requests for a singular processing node, the MCP triggers a deterministic conflict resolution protocol. This mechanism dynamically evaluates the priority-availability product to optimally reallocate pipeline resources and strictly prevent execution deadlock.

To address these limitations, a centralized controller with a shared policy network is introduced $\pi_{\theta }\left(a_{t}|s_{t}\right)$, where θ denotes the network parameters and $s_{t}$ denotes the unified input, integrating task queue information, resource states, and execution constraints. This centralized design reduces memory overhead and avoids policy homogenization through unified training, allowing the model to learn globally optimal allocation strategies. During decision making, the policy network outputs the action probability vector $P_{t}=\left[P_{1},P_{2},P_{3},P_{4}\right]$. The optimal assignment is determined as:

(8) $$a_{t}^{\mathrm{policy}}=\arg\max_{i}P_{i}$$

This deterministic decision mechanism eliminates ambiguity and enables global optimization. To address resource contention, a priority-aware conflict resolution mechanism can be defined as:

(9) $$a_{t}^{*}=\arg\max_{i}\left(q_{j}\cdot r_{i}\right)$$

where $q_{j}$ denote the priority of task *j*, and $r_{i}$ denote the availability of resource *i*.

This mechanism ensures that high-priority tasks are preferentially assigned to highly available resources. When the optimal resource is unavailable, tasks are dynamically reassigned to feasible idle agents, enabling efficient conflict resolution. The conflict resolution process also generates feedback signals for subsequent policy refinement. Furthermore, the MCP architecture establishes a closed-loop scheduling mechanism. The execution layer returns the feedback vector at time step *t*, which can be defined as:

(10) $$f_{t}=\left[u_{t},m_{t},h_{t}\right]$$

where $u_{t}$ represents resource utilization, $m_{t}$ represents task progress, and $h_{t}$ represents agent status. The global state transition is defined as:

(11) $$s_{t+1}=F\left(s_{t},a_{t},f_{t}\right)$$

### 3.4. Adaptive Policy Continuous Optimization

**Figure 5:**
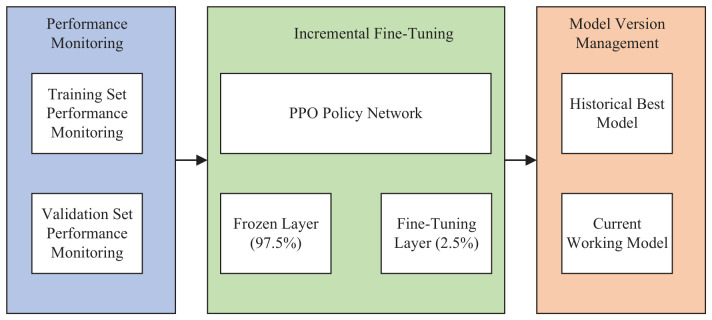
Illustration of the verification-driven adaptive policy continuous optimization mechanism. Different background colors are used to visually distinguish the distinct functional stages of the optimization process.

In SAR image interpretation tasks, environmental dynamics exhibit strong non-stationarity, including abrupt noise variation and emerging target categories, which may lead to policy degradation after deployment. To address this issue, a validation-driven adaptive policy optimization mechanism is developed. The proposed framework establishes a closed-loop optimization process consisting of performance monitoring, trigger evaluation, incremental update, and rollback protection, enabling continuous adaptation of the policy during operation, as illustrated in Figure 5. Specifically, the workflow initiates with a dual-track performance monitoring module that continuously evaluates both training set and validation set metrics to detect potential operational degradation. Upon satisfying specific trigger conditions, the system activates an incremental fine-tuning protocol on the Proximal Policy Optimization network. To ensure algorithmic resilience and prevent representational degradation, the architecture strategically freezes 97.5% of the basal network layers, restricting active parameter updates exclusively to the remaining 2.5% fine-tuning layer. Furthermore, a robust model version management system is integrated to enforce strict execution safety. This component maintains a secure repository of the historical best model, facilitating deterministic rollback protection should the current working model exhibit suboptimal adaptation to the fluctuating environment.

#### 3.4.1. Performance Monitoring and Trigger Conditions

To comprehensively assess policy stability during both training and deployment, a unified performance metric is introduced by jointly considering matching accuracy, scheduling latency, and interpretation efficiency and is continuously monitored over the training and validation stages; once the variation of training performance remains sufficiently small over several consecutive iterations, the model is regarded as entering a pre-convergent state, which serves as the basis for subsequent trigger-driven adaptation. A unified performance metric is defined as:

(12) $$J_{\theta }=\lambda_{1}{\mathrm{Acc}}_{\theta }-\lambda_{2}{\mathrm{Delay}}_{\theta }+\lambda_{3}{\mathrm{Eff}}_{\theta }$$

where Acc denotes matching accuracy, Delay represents scheduling latency, and Eff corresponds to interpretation efficiency. The weighting coefficients are empirically established as $\lambda_{1}=0.5$, $\lambda_{2}=0.3$, and $\lambda_{3}=0.2$. This specific configuration is designed to prioritize task-resource matching accuracy, which is paramount for downstream interpretation fidelity. Simultaneously, it imposes a disproportionate penalty on scheduling latency to strictly satisfy the real-time execution constraints inherent to airborne systems, achieving a robust operational trade-off.

To ensure that policy updates are activated only when necessary, two trigger mechanisms are introduced, namely performance degradation and distribution shift, where the former corresponds to a significant decline in validation performance relative to the historical best result, and the latter corresponds to validation loss exceeding its statistical stability range, either of which initiates the adaptive optimization process. The training and validation performance are recorded as $J_{\mathrm{train}}^{t},J_{\mathrm{val}}^{t}$. Convergence is determined by $|J_{\mathrm{train}}^{t}-J_{\mathrm{train}}^{t-k}|<\varepsilon$. Performance degradation trigger can be defined as $J_{\mathrm{val}}^{t}<J_{\mathrm{best}}\left(1-\delta \right)$. Distribution shift trigger can be defined as $L_{\mathrm{val}}^{t}>\mu_{L}+1.5\sigma_{L}$. The adaptive optimization process is activated when either condition is satisfied.

#### 3.4.2. Layered Freezing and Incremental Fine-Tuning

To reduce the computational burden of full retraining and avoid catastrophic forgetting, the policy parameters are decomposed into frozen and trainable subsets, where the underlying feature extraction parameters remain fixed and only a small set of high-sensitivity parameters associated with action generation and value estimation is incrementally updated, thereby enabling rapid environmental adaptation while preserving previously learned knowledge. The policy parameter set is decomposed as $\theta =\{\theta_{\mathrm{frozen}},\theta_{\mathrm{tune}}\}$. The frozen parameters remain unchanged $\theta_{\mathrm{frozen}}\leftarrow \theta_{\mathrm{frozen}}$. The trainable parameters are updated by $\theta_{\mathrm{tune}}\leftarrow \theta_{\mathrm{tune}}-\eta \nabla_{\theta_{\mathrm{tune}}}L\left(\theta \right)$. This mechanism enables efficient adaptation while preserving previously learned knowledge.

#### 3.4.3. Model Update and Rollback

To guarantee the safety of online optimization, a performance-driven model update criterion is adopted, under which a newly adapted model is accepted only when its validation performance exceeds that of the historical best model; otherwise, the parameters are immediately rolled back to the best preserved state, ensuring that adaptation never introduces performance degradation. The model update criterion is defined as $J_{\mathrm{val}}\left(\theta_{\mathrm{new}}\right)>J_{\mathrm{best}}\left(1+\varepsilon \right)$. The overall update process is formulated as $\theta_{t+1}=\mathrm{Update}\left(\theta_{t},D_{\mathrm{val}}\right)$, which establishes a validation-driven closed-loop optimization framework to ensure robust and stable policy adaptation under dynamic environments. The complete execution logic of the proposed framework, including orchestration, MCP scheduling, and continual adaptation, is summarized in Algorithm 1.
**Algorithm 1** MCP-Based Centralized Collaborative Scheduling and Adaptive Optimization**Require:** Agent resource state *A*, Task attributes *T*, Environmental parameters *E*, Initial policy parameters $\theta =\{\theta_{frozen},\theta_{tune}\}$**Ensure:** Optimal task allocation $a_{t}^{*}$, Updated policy parameters $\theta_{t+1}$1:**Phase 1: Multi-Source Data Fusion & Orchestration**2:Construct unified state representation: $S=\omega_{A}A⊕\omega_{T}T⊕\omega_{E}E$3:Calculate assignment probability vector: $\pi_{\theta }\left(S\right)=P$4:Determine preliminary assignment: $a_{t}^{Policy}=\arg\max_{i}P_{i}$5:**Phase 2: MCP Centralized Scheduling & Execution**6:**if** resource contention occurs among agents **then**7:   Resolve conflict using priority and availability: $a_{t}^{*}=\arg\max_{j}\left(q_{j}\cdot r_{i}\right)$8:**else**9:   $a_{t}^{*}=a_{t}^{Policy}$10:**end if**11:Execute task allocation $a_{t}^{*}$12:Execution layer returns feedback vector: $f_{t}=\left[u_{t},m_{t},h_{t}\right]$13:Update global state transition: $s_{t+1}=F\left(s_{t},a_{t},f_{t}\right)$14:**Phase 3: Adaptive Policy Continuous Optimization**15:Evaluate unified performance metric: $J_{\theta }=\lambda_{1}{\mathrm{Acc}}_{\theta }-\lambda_{2}{\mathrm{Delay}}_{\theta }+\lambda_{3}{\mathrm{Eff}}_{\theta }$16:**if** performance degradation $J_{val}^{t}<J_{best}\left(1-\delta \right)$
**or** distribution shift $L_{val}^{t}>\mu_{L}+1.5\sigma_{L}$ **then**17:   *// Incremental Fine-Tuning Mechanism Triggered*18:   Keep base feature extraction parameters unchanged: $\theta_{frozen}\leftarrow \theta_{frozen}$19:   Update trainable parameters via gradient descent: $\theta_{tune}\leftarrow \theta_{tune}-\eta \nabla_{\theta_{tune}}L\left(\theta \right)$20:   *// Model Update and Rollback Protection*21:   **if** $J_{val}\left(\theta_{new}\right)>J_{best}\left(1+\epsilon \right)$ **then**22:      Accept adapted model: $\theta_{t+1}=\mathrm{Update}\left(\theta_{t},D_{val}\right)$23:   **else**24:      Rollback parameters to historical best preserved state25:   **end if**26:**end if**27:**return** Optimal assignment $a_{t}^{*}$ and optimized policy $\theta_{t+1}$

## 4. Results

In this section, we first detail the experimental environment and parameter settings. Subsequently, we present comprehensive simulation results, including comparative evaluations and ablation studies, to demonstrate the effectiveness and superiority of the proposed multi-agent collaborative scheduling framework.

### 4.1. Parameter Settings

The experimental environment was established on a 64-bit operating system equipped with an Intel Core i7-8565 processor operating at 1.8 GHz, an NVIDIA MX150 graphics unit, 8 GB of system memory, and a 512 GB solid-state drive. Anaconda version 5.2 was used to manage the experimental environment. PyTorch version 2.10.0 served as the core deep learning framework. NumPy version 2.2.6 and Pandas version 2.3.3 were utilized for numerical computation and data manipulation. Visualization tasks were performed using Matplotlib version 3.10.8. To ensure the rigorous reproducibility of the proposed framework and facilitate future benchmark comparisons, the critical hyperparameters governing the PPO training phase are comprehensively detailed in Table 5 in strict alignment with standard reinforcement learning reporting protocols.

### 4.2. Evaluation Metrics

To comprehensively evaluate the effectiveness of the proposed multi-agent scheduling framework under SAR interpretation scenarios, three metrics are adopted, including task–agent matching accuracy, average scheduling delay, and average collaborative interpretation throughput.

#### 4.2.1. Task–Agent Matching Accuracy

Task–agent matching accuracy measures the consistency between the predicted agent assignment and the ground-truth task label, reflecting the correctness of task–resource orchestration. It can be defined as:

(13) $$\mathrm{ACC}=\frac{1}{N_{\mathrm{test}}}\sum_{i=1}^{N_{\mathrm{test}}}I\left(a_{i}=a_{i}^{*}\right)\times 100\%$$

where $N_{\mathrm{test}}$ denotes the number of samples in the test set, $a_{i}$ represents the predicted agent index for the *i*-th sample, and $a_{i}^{*}$ denotes the corresponding ground-truth label. The indicator function I(·) equals one when the prediction matches the ground truth and zero otherwise. The label space is defined over four agent types corresponding to detection, preprocessing, interpretation, and intelligence generation.

#### 4.2.2. Average Scheduling Delay

Average scheduling delay evaluates the time cost from task arrival to the initiation of execution, which reflects the responsiveness of the scheduling mechanism. It can be defined as:

(14) $$D=\frac{1}{N_{\mathrm{test}}}\sum_{i=1}^{N_{\mathrm{test}}}d_{i}$$

where $d_{i}$ denotes the scheduling delay of the *i*-th sample. To simulate realistic scheduling behavior, delay values are assigned according to decision correctness. Correct decisions are associated with lower delay values within a predefined range, while incorrect decisions incur higher delay values, thus capturing the impact of scheduling errors on system responsiveness.

#### 4.2.3. Average Collaborative Interpretation Throughput

Average collaborative interpretation throughput is measured in frames per second and reflects the overall processing efficiency and real-time capability of the system. It can be defined as:

(15) $$F=\frac{1}{N_{\mathrm{test}}}\sum_{i=1}^{N_{\mathrm{test}}}f_{i}$$

where $f_{i}$ denotes the processing throughput of the *i*-th sample. Higher throughput values correspond to more efficient collaborative processing. Similar to the delay modeling, correct decisions yield higher throughput, whereas incorrect scheduling decisions result in reduced processing efficiency.

#### 4.2.4. Selection of Comparative Methods

To validate the effectiveness of the proposed scheduling framework, extensive comparative experiments are conducted against several representative methods, including MAPPO [21], HAPPO [22], DQN [23], MLP [24], Transformer-based scheduling [25], and a conventional centralized scheduling algorithm [26]. All compared methods are implemented under the same experimental environment, dataset, and evaluation metrics, and their parameters are carefully tuned to ensure a fair comparison.

Specifically, MAPPO is a multi-agent reinforcement learning method based on the actor–critic paradigm, where a centralized critic evaluates the global state while decentralized actors execute policies, and a shared policy network is optimized using advantage estimation [21]. HAPPO extends this framework to heterogeneous agent settings by allowing different agents to adopt distinct network architectures and employs a sequential update strategy to optimize heterogeneous policies [22].

DQN represents value-based reinforcement learning, where action selection is achieved through approximation of the Q-function using a dual-network structure combined with an exploration–exploitation mechanism [23]. MLP serves as a supervised learning baseline with a standard feedforward architecture, directly mapping input features to task labels without considering agent collaboration or sequential decision dependencies, thereby providing an upper bound under ideal classification assumptions [24].

The Transformer-based scheduler models the task allocation problem using self-attention mechanisms, encoding both task features and agent characteristics into a sequence representation, and capturing complex interactions through multi-head attention while leveraging positional encoding to describe agent heterogeneity [25].

In addition, a conventional centralized scheduling method is included as a non-learning baseline, where task assignment is determined by predefined heuristic rules without data-driven optimization [26].

The proposed method adopts a PPO-based policy optimization framework integrated with a centralized MCP scheduling architecture. It incorporates multi-source information fusion into a unified state representation and employs a clipped policy update mechanism to stabilize training. Residual connections and normalization techniques are introduced to enhance feature extraction capability and convergence behavior. Furthermore, the framework integrates task–resource orchestration and feedback-driven optimization modules, forming a complete closed-loop scheduling system.

#### 4.2.5. Comparative Results and Analysis

Table 6 presents the performance of all methods in terms of task–agent matching accuracy, scheduling delay, and collaborative interpretation throughput.

**Figure 6:**
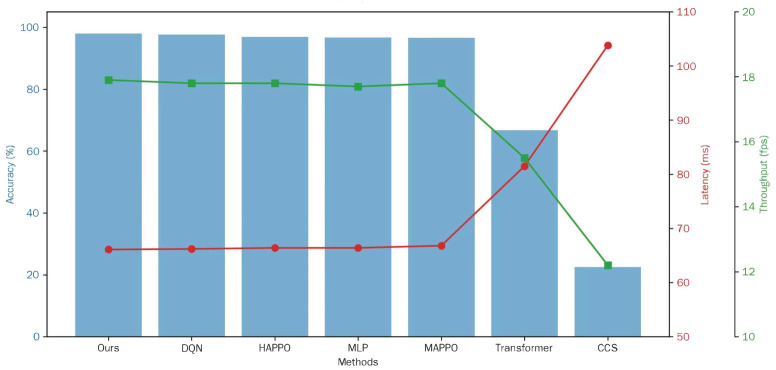
Performance comparison of different scheduling methods in terms of accuracy, latency, and throughput.

It can be observed from Table 6 and Figure 6 that the compared methods exhibit a clear performance hierarchy. The first group includes the proposed method, DQN, HAPPO, MAPPO, and MLP, all of which achieve accuracy higher than 96 percent, maintain scheduling delay within a narrow range around 66 milliseconds, and provide throughput no lower than 16 frames per second, thereby satisfying real-time requirements. The second group corresponds to the Transformer-based scheduler, whose accuracy significantly decreases to 66.75 percent, accompanied by increased latency and reduced throughput, resulting in failure to meet real-time constraints. The third group is represented by the conventional CCS method, which shows extremely low accuracy and poor efficiency, indicating the limitations of rule-based scheduling approaches.

The proposed method achieves the best overall performance across all evaluation metrics, with an accuracy of 97.98 percent, an average delay of 66.1 milliseconds, and a throughput of 17.9 frames per second. Compared with multi-agent reinforcement learning methods, the proposed approach improves accuracy by 1.15 percent over MAPPO and 0.90 percent over HAPPO. This improvement is attributed to the clipped policy update mechanism of PPO, which effectively stabilizes training, as well as the integration of residual connections and normalization techniques that enhance feature representation. In contrast, the heterogeneous structure in HAPPO increases optimization complexity without yielding expected performance gains.

When compared with the value-based method DQN, both methods achieve comparable accuracy. However, the proposed policy-based approach demonstrates more stable decision behavior and lower memory consumption, making it more suitable for resource-constrained onboard environments.

From the perspective of real-time performance, a strong correlation can be observed between matching accuracy, scheduling delay, and throughput. Methods with higher accuracy consistently achieve lower latency and higher throughput. In contrast, methods with poor decision accuracy frequently trigger high-delay and low-efficiency execution branches, leading to degraded overall performance.

Overall, the experimental results demonstrate that the proposed PPO-based centralized scheduling framework provides significant advantages in SAR interpretation scenarios. Compared with the conventional CCS method, the proposed approach improves accuracy by 75.48 percent and reduces scheduling delay by 36.3 percent, satisfying practical real-time constraints. Compared with multi-agent reinforcement learning methods, the simplified centralized PPO formulation achieves superior performance in the considered task allocation scenario, indicating that complex multi-agent coordination mechanisms are not always necessary. Furthermore, the substantial performance gap between the proposed method and the Transformer-based approach confirms the effectiveness of modeling the scheduling problem as a decision process rather than a sequence generation task.

#### 4.2.6. Ablation Experiment Design

To evaluate the contribution of each major component in the proposed framework, an ablation study was conducted by removing one module at a time from the full model while keeping all other experimental settings unchanged. Three ablation variants were constructed, corresponding to the removal of the multi-source information orchestration module, the MCP-based centralized collaborative scheduling module, and the adaptive policy continual optimization module.

For Model-1, the multi-source information orchestration module was removed by replacing the original attention-based dynamic fusion strategy with fixed-weight averaging. The adaptive fusion formulation $S_{\mathrm{fusion}}=\omega_{A}A+\omega_{T}T+\omega_{E}E$ was simplified by setting $\omega_{A}=\omega_{T}=\omega_{E}=1/3$. In addition, dynamic state information, such as real-time workload and energy consumption, was excluded from the decision process.

For Model-2, the MCP-based centralized collaborative scheduling mechanism was removed and replaced with a distributed independent decision-making scheme. The global task queue and conflict resolution mechanism were disabled, and the four functional agents independently produced local decisions, which were then combined through voting or probabilistic aggregation.

For Model-3, the adaptive policy continual optimization module was removed by disabling the online incremental fine-tuning mechanism after the initial training stage. Model parameters remained unchanged during deployment, and no human feedback was introduced for policy updating.

#### 4.2.7. Ablation Results and Discussion

**Figure 7:**
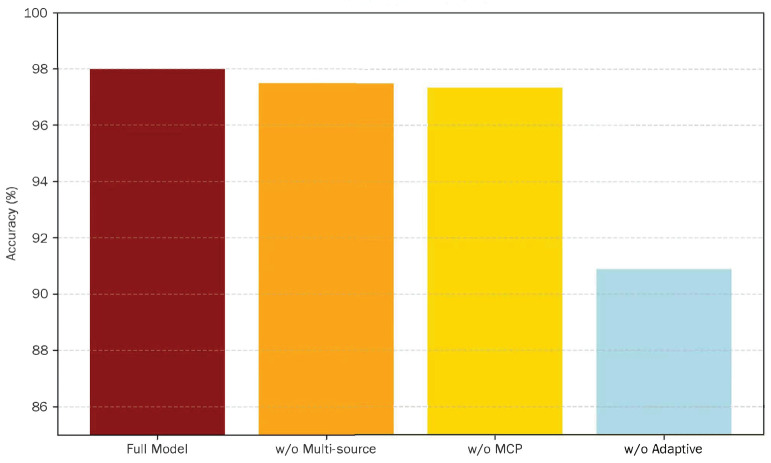
Ablation study results demonstrating the impact of each core module on task-agent matching accuracy.

The ablation study results are summarized in Table 7 and visually compared in Figure 7. The full model achieves the best overall performance, yielding an accuracy of 97.98%, a scheduling latency of 66.1 ms, and an interpretation throughput of 17.9 fps. To quantitatively evaluate the contribution of each core component, three ablated variants are constructed by removing the multi-source information orchestration module, the MCP-based centralized collaborative scheduling module, and the adaptive policy continual optimization module, respectively.

## 5. Discussion

The results demonstrate that all three modules contribute positively to system performance, while their impacts exhibit different magnitudes. Specifically, removing the MCP-based centralized collaborative scheduling module leads to a decrease in accuracy from 97.98% to 97.33% (−0.67%). This degradation indicates that centralized coordination plays a critical role in maintaining global information consistency and resolving resource conflicts among multiple agents. Without MCP, the system degenerates into a distributed decision-making paradigm, where the lack of global awareness limits cooperative efficiency and overall scheduling optimality.

The removal of the multi-source information orchestration module results in a moderate accuracy drop to 97.48% (−0.51%). This observation suggests that adaptive fusion of heterogeneous information, including device states, task characteristics, and environmental parameters, improves task–resource matching under dynamic conditions. Compared with fixed-weight fusion, the proposed dynamic weighting strategy enhances robustness against environmental uncertainty and scenario variability, although its contribution is relatively smaller compared with other components.

The most significant performance degradation is observed when the adaptive policy continual optimization module is removed. In this case, the accuracy decreases sharply to 90.88% (−7.25%), accompanied by increased scheduling latency (69.3 ms) and reduced interpretation throughput (17.4 fps). This result highlights that continual policy adaptation is essential for maintaining long-term performance stability. Specifically, the adaptive optimization module achieves algorithmic resilience by decoupling generalized feature extraction from local policy execution. In the presence of sudden domain shifts, particularly the abrupt electromagnetic interference intrinsic to SAR acquisition, the frozen basal layers constituting 97.5% of the network parameters preserve the foundational target representations. This architectural design effectively averts representational degradation and prevents over-adaptation to transient disturbances. Concurrently, the active fine-tuning layer rapidly recalibrates the action probability distribution, thereby ensuring continuous structural adaptation to non-stationary noise profiles while sustaining overarching scheduling stability.

The ablation results validate both the effectiveness and the complementarity of the proposed modules. The MCP-based centralized scheduling mechanism serves as the fundamental backbone for global coordination, while multi-source information fusion and continual policy optimization further enhance feature representation and adaptive decision-making capabilities. Their joint integration forms a coherent framework that ensures high accuracy, low latency, and strong robustness for SAR image interpretation in dynamic scenarios.

Furthermore, regarding practical deployment feasibility, the proposed framework is specifically designed for resource-constrained airborne environments from both algorithmic and system perspectives. Algorithmically, the lightweight PPO-based policy network adopts a compact architecture, ensuring low computational complexity and a minimal memory footprint. Concurrently, the centralized MCP scheduling mechanism significantly reduces inter-agent communication overhead, a critical factor for onboard real-time execution. From a system perspective, the achieved scheduling latency of 66.1 ms and throughput of 17.9 fps demonstrate that the proposed method strictly satisfies real-time processing requirements under typical airborne mission constraints. These operational characteristics confirm that the framework is highly compatible with contemporary edge computing platforms equipped with embedded GPUs or AI accelerators. Future research will focus on migrating the proposed framework to edge-optimized AI runtimes such as TensorRT, evaluating its empirical performance on airborne platforms under realistic power, payload, and communication constraints.

## 6. Conclusions

To address the challenges of scheduling latency, suboptimal task–resource matching, and limited coordination efficiency in dynamic SAR image interpretation scenarios, this paper proposed an MCP-based centralized multi-agent collaborative scheduling framework for airborne SAR autonomous interpretation. The proposed system integrates task–resource intelligent orchestration, centralized collaborative scheduling, interpretation model adaptation, and adaptive policy continual optimization into a unified end-to-end pipeline. Extensive experiments conducted on the SARCAP dataset, including both comparative and ablation studies, demonstrate the effectiveness of the proposed approach. The framework achieves a task–agent matching accuracy of 97.98%, a scheduling latency of 66.1 ms, and an interpretation throughput of 17.9 fps, consistently outperforming representative baselines such as MAPPO, HAPPO, and conventional centralized scheduling methods. The results indicate that the proposed method enables efficient coordination across sensing, preprocessing, interpretation, and intelligence generation agents, thereby effectively addressing the multi-agent collaborative scheduling problem in SAR image interpretation and providing a robust solution for airborne intelligent processing systems. Future work will focus on enhancing real-time resource perception, improving multi-task concurrent scheduling capability, and exploring lightweight learning strategies under limited labeled data.

## Figures and Tables

**Table 1:** SARCAP dataset scene features and computational parameters.

Scene Type	Keywords	Scene Complexity	Computational Demand
Port	harbor, port, dock, terminal, aquatic transport	80	90
Wharf	wharf, berth, pier, maritime commerce	85	95
Nearshore	coastal, shore, nearshore	60	70
Open Ocean	ocean, sea, open water, marine	40	50

**Table 2:** Task attribute configuration for multi-agent scheduling.

Task Type	Keywords	Agent Class	Priority	Accuracy Requirement	Time Constraint (ms)
Interpretation	identify, recognize, locate, coordinate, feature, detect, find	Interpretation Agent	4	86–94	50–75
Detection	detection, quality, screen, capture, collect	Detection Agent	3	60–78	90–120
Preprocessing	denoise, register, enhance, process, pretreat	Preprocessing Agent	2	75–85	70–95
Intelligence Generation	integrate, analyze, report, information, summary	Intelligence Agent	1	95–99	30–55

**Table 3:** Environmental parameter ranges.

Parameter	Range	Description
Noise Level	10–90	Speckle noise intensity and interference level
Scene Complexity	20–95	Background clutter complexity
Motion Stability	60–99	Platform or target motion stability

**Table 4:** Standardized dataset schema.

Column Name	Data Type	Description
image_path	STRING	Path to SAR image file
target_labels	STRING	Object category and bounding box annotations
caption	STRING	Natural language description of the image
task_type	STRING	Assigned task category
priority	STRING	Task priority level
accuracy_req	INTEGER	Required accuracy level
time_limit_ms	INTEGER	Latency constraint in milliseconds
env_noise	INTEGER	Environmental noise level
scene_complexity	INTEGER	Scene complexity index
motion_stable	INTEGER	Motion stability index
split	STRING	Training or testing split

**Table 5:** Key training hyperparameters of the Proximal Policy Optimization algorithm.

Hyperparameter	Symbol	Value
State Dimension	|S|	10
Hidden Layer Size	-	64
Learning Rate (Actor/Critic)	$\alpha_{a}/\alpha_{c}$	$3\times 10^{-4}/1\times 10^{-3}$
Batch Size	*B*	256
Discount Factor	γ	0.99
Clip Threshold	ϵ	0.2

**Table 6:** Performance comparison of different methods.

Method	Acc (%)	D (ms)	F (fps)
Ours (PPO Agent)	97.98	66.1	17.9
DQN (Value-Based RL)	97.60	66.2	17.8
HAPPO (Heterogeneous A2C)	96.85	66.4	17.8
MLP (Supervised)	96.65	66.4	17.7
MAPPO (Multi-Agent A2C)	96.60	66.8	17.8
Transformer (Attention)	66.75	81.5	15.5
CCS (Conventional)	22.50	103.8	12.2

**Table 7:** Ablation study results of the proposed method performance.

Model Configuration	Accuracy (%)	Latency (ms)	Throughput (fps)	Performance Drop
**Full Model**	**97.98**	**66.1**	**17.9**	–
Without Multi-Source Information Orchestration	97.48	66.2	17.8	↓ 0.51%
Without MCP-Based Centralized Scheduling	97.33	66.4	17.8	↓ 0.67%
Without Adaptive Policy Continual Optimization	90.88	69.3	17.4	↓ 7.25%

**Note:** Bold formatting highlights the best performance achieved by the full proposed model. The ↓ symbol indicates a performance decrease relative to the full model.

## Data Availability

The experiments in this study were conducted based on the De-SAR detection subset of the public SARCAP dataset. The generated training and testing samples for scheduling simulation are not publicly available due to the specific task design of this work. The processed experimental data are available from the corresponding author upon reasonable request.
